# Calcium Phosphate Spacers for the Local Delivery of Sitafloxacin and Rifampin to Treat Orthopedic Infections: Efficacy and Proof of Concept in a Mouse Model of Single-Stage Revision of Device-Associated Osteomyelitis

**DOI:** 10.3390/pharmaceutics11020094

**Published:** 2019-02-22

**Authors:** Ryan P. Trombetta, Mark J. Ninomiya, Ihab M. El-Atawneh, Emma K. Knapp, Karen L. de Mesy Bentley, Paul M. Dunman, Edward M. Schwarz, Stephen L. Kates, Hani A. Awad

**Affiliations:** 1 Department of Biomedical Engineering, University of Rochester, Rochester, NY 14642, USA; ialatawn@u.rochester.edu (I.M.E.-A.); Edward_Schwarz@URMC.Rochester.edu (E.M.S.); Hani_Awad@URMC.Rochester.edu (H.A.A.); 2 Center for Musculoskeletal Research, University of Rochester, Rochester, NY 14642, USA; Mark_Ninomiya@URMC.Rochester.edu (M.J.N.); Emma_Gira@URMC.Rochester.edu (E.K.K.); Karen_Bentley@URMC.Rochester.edu (K.L.d.M.B.); Stephen.Kates@vcuhealth.org (S.L.K.); 3 Department of Microbiology and Immunology, University of Rochester, Rochester, NY 14642, USA; Paul_Dunman@URMC.Rochester.edu; 4 Department of Pathology & Laboratory Medicine, University of Rochester, Rochester, NY 14642, USA; 5 Department of Orthopedics, University of Rochester Medical Center, Rochester, NY 14642, USA; 6 Department of Orthopaedic Surgery, Virginia Commonwealth University School of Medicine, Richmond, VA 0153, USA

**Keywords:** osteomyelitis, *Staphylococcus aureus*, sitafloxacin, rifampin, calcium phosphate, 3D printing, drug delivery, 1-stage revision, PMMA

## Abstract

Osteomyelitis is a chronic bone infection that is often treated with adjuvant antibiotic-impregnated poly(methyl methacrylate) (PMMA) cement spacers in multi-staged revisions. However, failure rates remain substantial due to recurrence of infection, which is attributed to the poor performance of the PMMA cement as a drug release device. Hence, the objective of this study was to design and evaluate a bioresorbable calcium phosphate scaffold (CaPS) for sustained antimicrobial drug release and investigate its efficacy in a murine model of femoral implant-associated osteomyelitis. Incorporating rifampin and sitafloxacin, which are effective against bacterial phenotypes responsible for bacterial persistence, into 3D-printed CaPS coated with poly(lactic co-glycolic) acid, achieved controlled release for up to two weeks. Implantation into the murine infection model resulted in decreased bacterial colonization rates at 3- and 10-weeks post-revision for the 3D printed CaPS in comparison to gentamicin-laden PMMA. Furthermore, a significant increase in bone formation was observed for 3D printed CaPS incorporated with rifampin at 3 and 10 weeks. The results of this study demonstrate that osteoconductive 3D printed CaPS incorporated with antimicrobials demonstrate more efficacious bacterial colonization outcomes and bone growth in a single-stage revision in comparison to gentamicin-laden PMMA requiring a two-stage revision.

## 1. Introduction

Orthopedic implants, most commonly associated with musculoskeletal injuries and joint arthroplasty, are highly susceptible to recurrent bacterial bone infections known as osteomyelitis. Infection rates range between 7.8–23.6% for internal stabilization and 0.76–1.28% for joint arthroplasties, which result in over 100,000 infected orthopedic devices each year in the United States [1,2,3,4,5,6]. Consequently, once a patient is infected, there is a 40–42% treatment failure rate, resulting in recurrence of infection [7,8]. Due to the chronic nature of implant-associated infections, costs can exceed over $100,000 per patient [9]. 

The socioeconomic burden of implant-associated osteomyelitis is attributed to the inadequate ability of current clinical approaches to eradicate pathogens, predominantly *Staphylococcus aureus*, which have a predisposition to colonize implant surfaces [10]. The current gold standard for treating infected joint arthroplasties is a two-stage revision surgery utilizing surgical debridement and complete hardware exchange. This procedure accounts for 60% of all performed surgical interventions for prosthetic joint infections, while ~36% are single-stage revisions, and ~4% are amputations [11]. During a two-stage revision, the infected hardware is removed, necrotic bone and any pathological tissue is debrided, pulsatile lavage is performed to irrigate the wound and then a temporary antibiotic-laden poly (methyl methacrylate) (PMMA) bone cement spacer or beads are inserted into the joint [12,13,14]. Systemic antibiotics are administered and after a period of 2–10 weeks a revision surgery is performed, in which the bone cement beads are removed and a new prosthesis is installed [15,16]. In cases of infection after fracture fixation, the same surgical algorithm is followed, except a bone graft and new stabilization hardware is inserted during the revision surgery to permit bone healing [17]. The local delivery of antibiotics via PMMA cement is implemented in order to deliver high concentrations of drug to the infected tissues, which cannot be achieved by systemic administration due to pharmacokinetic distribution, bioavailability, and toxicity limitations. However, *S. aureus* colonies are still able to survive this clinical treatment and contribute to the recurrence of infection [18,19]. 

*Staphylococcus aureus* can enter an alternative growth state, forming a bacterial community capable of producing an extracellular matrix consisting of polysaccharides and proteins, known as the biofilm. Orthopedic implants are predisposed to the formation of biofilm because once implanted, the coating of host proteins facilitate the attachment, colonization, and maturation of bacterial communities [20]. Once established, biofilm enables the bacteria within to evade host immune defenses and survive antibiotic treatment providing a physical barrier limiting drug diffusion and interaction with host immune cells [21,22,23]. *Staphylococcus aureus* can also transform into an alternative phenotype known as small colony variants (SCV). Small colony variants have an altered and reduced metabolism, leading to reduced susceptibility to local and systemic antibiotics, such as aminoglycosides [24]. Additionally, SCV have the ability to persist intracellularly enabling evasion from host immune cells [25,26]. Small colony variants are often overlooked in recurrent infections: however, when properly identified, 34% of prosthetic joint infections have SCV [27]. Lastly, SCV is an unstable phenotype that has the ability to revert back to the virulent normal colony phenotype, providing an additional avenue for the recurrence of osteomyelitis [28].

Both biofilm and SCV contribute to the chronic nature of implant-associated infections and explain the recurrence of infection after dormancy, which could last as long as 80 years [29]. Compounding these phenotypes of *S. aureus* is the inconsistent and unsatisfactory performance of antibiotic-impregnated PMMA cement. Studies document highly variable drug release kinetics and demonstrate a burst-release within the first 24 h, followed by a dramatic reduction resulting in no further drug elution past the first week [30,31]. This bolus release is not adequate for infection management when spacers remain in vivo for up to 10 weeks [16]. The poor release enables the emergence of resistant phenotypes and the depleted spacer provides a nidus for biofilm formation [32,33,34]. Additionally, only select antibiotics can be mixed with PMMA without adversely affecting its polymerization, such as gentamicin, tobramycin, or vancomycin [35]. However, none of the PMMA-compatible antibiotics are effective against biofilm [33]. Further, PMMA is not bioresorbable and hence, a revision surgery is necessary to remove the implant and insert a new prosthesis or bone graft to enable bone healing. Therefore, there is a clinical need to produce biocompatible and osteoconductive spacers, which can provide a vehicle for local drug delivery of effective antimicrobials and enable bone regeneration. Such a scaffold would eliminate the need for a revision surgery to remove the PMMA bone cement spacer, thus reducing the physical and financial burdens of treating implant-associated osteomyelitis.

At minimum, three critical design criteria must be considered when engineering an effective vehicle for drug delivery in the treatment of implant-associated osteomyelitis. These criteria include (1) compatibility with incorporating antimicrobials that are potent against a variety of *S. aureus* strains and phenotypes (i.e., biofilm and SCV), (2) surgical biocompatibility, and (3) osteoconductivity. Calcium phosphate scaffolds (CaPS) have been proven to provide both a biocompatible and osteoconductive scaffold providing a platform for drug delivery at both the research and commercial level [36,37,38,39]. Rifampin is effective in the management of orthopedic infections due to its proven potency against multiple strains of *S. aureus* and established biofilm [40], but rifampin resistance is easy to achieve and the agent is incompatible with PMMA bone cement due to its interference with the polymerization process [35]. Therefore, incorporation of rifampin in alternative biomaterials is an appealing strategy to overcome this caveat, yet would likely still suffer from resistance. Additionally, sitafloxacin has recently shown great promise in the context of orthopedic infections due to high potency against methicillin-susceptible *S. aureus* (MSSA), methicillin-resistant *S. aureus* (MRSA), *S. aureus* biofilm, and SCV [41]. Therefore, incorporation of both antibiotics is a promising strategy for local delivery drug deliver for management of osteomyelitis. Further, resistance to the combination of sitafloxacin and rifampin would likely be slow to develop. A recent in vivo study showcased the ability to incorporate both vancomycin and rifampin into 3D printed CaPS in the treatment of implant-associated osteomyelitis with promising results [36]. However, despite the concomitant local delivery of vancomycin and rifampin, the treatment failed in eradicating biofilm on the surface of the retained orthopedic hardware. Because implant retention is seldom performed, the utilization of an infection model that involves hardware exchange is more suited to evaluate new surgical and therapeutic approaches using clinically translatable outcomes. To that end, we recently developed a mouse model of implant-associated osteomyelitis that involves complete hardware exchange and demonstrated the persistence of the infection despite the insertion of an antibiotic-laden PMMA spacer [42]. 

The objective of this study is two-fold. We first set out to design a biocompatible and osteoconductive CaPS to achieve sustained release of antimicrobial drugs that are effective against biofilm and SCV. Subsequently, we investigated the efficacy of the antibiotic-laden CaPS in the management of implant-associated osteomyelitis and bone healing in a single-stage revision approach with complete hardware exchange. We hypothesize that osteoconductive CaPS designed for sustained release of rifampin and sitafloxacin will significantly ameliorate outcomes of implant-associated osteomyelitis compared to the clinically utilized PMMA spacers, and concomitantly enhance bone regeneration in a critical size femoral defect in a single-stage revision procedure.

## 2. Materials and Methods 

### 2.1. Fabrication of 3D Printed Antibiotic-Laden Calcium Phosphate Acaffolds (CaPS) and Poly (Methyl Methacrylate) PMMA Spacers

A commercial 3D printer, ZPrinter 450 (3D Systems; Andover, MA), was modified to print osteoconductive bone graft substitutes as previously described [39]. In short, a phosphoric acid (8.75%)-based binder solution supplemented with 0.25 wt % Tween80 is sprayed by thermal inkjets (HP11, Hewlett-Packard; Palo Alto, CA) to selectively bind a calcium phosphate powder bed consisting of α-tricalcium phosphate (α-TCP) (Innotere; Radebeul, Germany) and hydroxyapatite (HA) (Sigma–Aldrich; St. Louis, MO). The particle size distribution ranged from 40–70 microns at a α-tricalcium phosphate:hydroxyapatite (TCP:HA) ratio of 80:20. The binder liquid-to-powder ratio was set to 0.46 and the layer thickness to 89 µm in the ZPrint™ software. The dissolution–precipitation reaction between the acid and the ceramic particles produced dicalcium phosphate dehydrate (DCPD; brushite) cement phase, which binds the particles in the layer and in between the successive layers. We fabricated 3D printed elongated ellipse constructs (major diameter = 2.1 mm, minor diameter = 1.25 mm with a length of 3 mm) for surgical implantation. Rectangular prisms (4 × 1 × 14 mm) were also printed for flexure testing. Sitafloxacin (Musechem; Fairfield, NJ) and rifampin (Sigma–Aldrich; St. Louis, MO) were incorporated into the 3D printing process by direct manual and mechanical mixing of the drug into the biphasic CaP powder at 1 wt % on a shaker. CaPS were fabricated with either sitafloxacin (Sita_1%_; ~28 µg/scaffold), rifampin (Rif_1%_; ~33 µg/scaffold), or a combination of both (Rif_1%_ + Sita_1%_; ~28 sitafloxacin/scaffold and ~33 µg rifampin/scaffold). 

To achieve sustained drug release from CaPS, scaffolds were coated with two layers of drug-containing poly (D,L-lactide-co-glycolide) (PLGA; 50:50 L:G; MW 24-38 kDa; Sigma–Aldrich; St. Louis, MO). PLGA was first dissolved at 20 wt % in dichloromethane in combination with 40 mg/mL or 20 mg/mL of each respective drug. The CaPS were soaked in this solution for 1 h to adsorb the drug containing PLGA and then dried at 40°C in a vacuum chamber overnight. A second coat of drug-laden PLGA was applied in which 12.5 wt % PLGA was dissolved in DCM with 40 mg/mL or 20 mg/mL of each respective drug. CaPS were soaked again for 1 h and then dried overnight at 40°C in a vacuum chamber completing preparation of the composite scaffolds for implantation into the mice.

The PMMA bone cement spacers (Cobalt G-HV; DJO Global; Vista, Ca), with and without 1.25 wt % gentamicin, were prepared in accordance with the manufacturer’s guidelines. Cement spacers were shaped and cured into a 3-mm long cylinder (1.8-mm diameter) in a custom mold prior to implantation. 

### 2.2. Characterization of Antibiotic Release and Bioactivity

A Kirby–Bauer disc diffusion assay was performed to measure the bioactivity and functionality of incorporated drug in each 3D printed CaPS. Sitafloxacin and rifampin were eluted into phosphate buffered saline (PBS) from CaPS. Filter discs (7-mm diameter) were soaked in 1 mL of each eluted solution overnight at 4°C. The disks were then gently dabbed to dry and incubated on Tryptic Soy agar (TSA) plates inoculated with a bioluminescent strain of methicillin-sensitive *S. aureus* (Xen36; PerkinElmer, Inc.; Waltham MA) overnight at 37 °C. The diameter of the zones of inhibition were then measured the following day. The diameter of the disk was subtracted from the diameter of the zone of inhibition.

To measure the antibiotic release kinetics, CaPS were placed in Eppendorf tubes with 250 µL of PBS at 37 °C. The CaPS were transferred to new vials of PBS at 1, 2, 4, and 8 h and then once daily for 2 weeks. Release of the drugs were quantified by optical absorbance, 340 nm for sitafloxacin and 330 nm for rifampin using a plate reader (BioTek SynergyMx). The limit of quantification was 4.35 µg/mL for rifampin and 8.53 µg/mL for sitafloxacin for each respective spectrum. 

### 2.3. Biomechanical Properties of 3D Printed Antibiotic-Laden Calcium Phosphate Scaffolds Coated with PLGA

The flexural mechanical properties of the 3DP CaPS were determined by 3-point bending until the failure of of 3DP rectangular prisms using an Instron 8841 DynaMight™ Axial Testing System (Instron Corp.; Canton, MA, USA) with a 50 N load cell. A 10-mm support span was used, and the bars were flexed using a central loading tip in the displacement control at a rate of 1 mm/min. 

### 2.4. Animal and Surgical Procedures

All animal studies were performed in accordance with protocols approved by the University of Rochester’s Committee on Animal Resources. Female Balb/cJ mice (13–15 weeks old) were purchased from Jackson Research Labs (Bar Harbor, ME) and acclimated for 1 week prior to surgeries. 

A mouse femoral ostectomy model was used to assess the efficacy of 3D printed CaPS for implant-associated osteomyelitis as previously published [42]. Briefly, mice were anesthetized by a combination of ketamine (130 mg/kg) and xylazine (12 mg/kg) injected intraperitoneally. The right femur was exposed by a lateral approach blunt dissection and a titanium screw (0.3 mm diameter, 2 mm length; RISystems; Davos, Switzerland) inoculated with Xen36 (~2.5 × 10^6^ colony forming units (CFU)/mL) was inserted into the mid-diaphysis of the femur through the anterolateral surface. The soft tissue and skin were closed, and the screw remained in vivo for seven days to establish the infection. 

**Figure 1:**
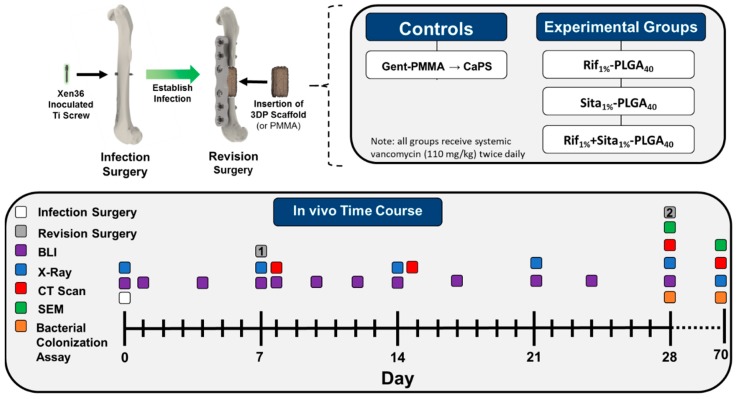
Schematic overview of the murine femoral ostectomy model used to assess 3D printed antibiotic-impregnated spacers for implant-associated osteomyelitis. First, a surgery was performed to establish an infection in the mouse’s femur. A titanium screw inoculated with bioluminescent Xen36 (~2.5 × 10^6^ CFU/mL) was inserted in the mid-diaphysis of a 13–15-week-old female Balb/cJ mouse. The infection was established for 7 days and then a revision surgery was performed in which the contaminated screw was removed and the femur was stabilized with a polyether ketone (PEEK) plate with 4 titanium screws. A 3-mm ostectomy is then performed to debride the infected mid-diaphysis of the femur. A poly(methyl methacrylate) (PMMA) cement spacer impregnated with gentamicin (1.25 wt %; Cobalt MV) or a 3D printed calcium phosphate scaffold (CaPS) with incorporated rifampin, sitafloxacin, or both is then inserted into the defect. Relapse in infection is continually monitored via bioluminescent imaging on days 1, 3, 5, 7, 12, 14, 17, 21, 24, and 28 days post-revision. The mouse was then euthanized 3 weeks post-revision surgery and the hardware, bone, cement spacer, and surrounding soft tissue is harvested for CFU analysis. *N* = 8/group. Long-term bone healing was also assessed 10 weeks post-revision in an identical cohort. In this study, the clinical control utilizing Gent-PMMA underwent a second revision surgery 3 weeks after the PMMA insertion, in which the Gent-PMMA spacers were removed and a 3D printed CaPS was inserted. Bone healing was assessed for 10 weeks post-revision. *N* = 8/group.

On day seven a complete hardware exchange revision surgery was performed on the infected femur. First, the contaminated screw was removed, and all visibly inflamed soft tissue was debrided. Next the bone was stabilized with a 6-hole radiolucent polyether ketone (PEEK) plate and 4 titanium screws inserted in the outermost proximal and distal holes (RISystems; Davos, Switzerland). A cutting guide was then attached to the plate and a 3-mm transverse ostectomy flanking the original location of the infected screw was performed using a 0.67-mm Gigli saw (RISystems; Davos, Switzerland) and the debrided bone was removed. The defect was then irrigated by lavage with PBS. In order to prevent septic loosening, the proximal and distal ends of the plate were fastened with a 5–0 nylon monofilament suture. The antibiotic-laden spacer (Gent-PMMA or CaPS with incorporated drug) was then inserted into the defect and secured in cerclage fashion using a 6–0 nylon braided suture. The wound was then sutured closed, and the mouse was allowed to heal, while receiving systemic doses of vancomycin (110 mg/kg subcutaneously twice daily) for 21 days post-revision. Mice were allotted to two studies; (1) a short-term time course with the objective to assess infection management 21 days post-revision surgery, and (2) a long-term healing time course to assess bone healing lasting 10 weeks post-revision surgery (Figure 1). In the long-term time course, Gent-PMMA mice underwent an additional revision surgery at 3 weeks removing the cement spacer and replaced with a CaPS to allow healing for an additional 10 weeks. The sample size was 8 mice per group.

### 2.5. Radiographic Imaging and Quantification

Planar X-ray images (LX-60 X-ray Cabinet, Faxitron Bioptics LLC; Tuscon, AZ, USA) were acquired (26 kV for 5 seconds) immediately post-surgery and every 3 weeks post-operation for up to 10 weeks. In vivo µ-CT scans (isotropic resolution of 17.5 µm) were performed post-revision surgery and at 3- and 10-week post-revision surgery. Scans were acquired with an energy of 55 kV, intensity of 145 µA, and 300 ms integration time. Volumetric µ-CT analysis was performed to measure bone growth as previously described [43]. Briefly, a constant volume of interest (VOI) was selected for all specimens from two-dimensional images spanning a length of 4.75 mm in between the two proximal most screws. A thresholding intensity corresponding to 50% of the peak intensity frequency was implemented to select bone voxels for each specimen. Bone volume was manually segmented in the VOI including both the callus and bone ingrowth into the CaPS. The axial length (4.75 mm) of the region of interest for measuring ingrowth was kept constant for each specimen. Quantification of bone growth in the Gent-PMMA groups was calculated as the difference between the second revision when the CaPS was inserted, and the end time point 10 weeks later.

### 2.6. Bioluminescent Imaging (BLI) of the Bacterial Burden

In vivo longitudinal bioluminescence was performed using an IVIS Spectrum imaging system (PerkinElmer, Inc.; Waltham, MA, USA) with an automatic exposure time. Average bioluminescence radiance was calculated by Living Image software (Version 3.2, PerkinElmer, Inc., Hopkinton, MA, USA, 2009) within a fixed region of interest around the infected thigh.

### 2.7. Serum C-Reactive Protein (CRP) 

Blood was collected on day 28 using a submandibular bleeding technique. A 5-mm lancet was used to puncture the retro-orbital vein and blood was collected in an Eppendorf tube. After collection, blood was allowed to clot for 1 h at room temperature before centrifuging for 20 minutes at 20,00× *g*. Serum was then collected and stored at −20 °C. Serum CRP levels were quantified using a mouse-CRP Quantikine ELISA kit (R&D System, Minneapolis, MN, USA) in accordance with the manufacturer’s instructions. 

### 2.8. Scanning Electron Microscopy (SEM) of Harvest Titanium Screws

Scanning electron microscopy (SEM) of titanium screws excised and fixed in 2.5% glutaraldehyde, post-fixed in 1.0% osmium tetroxide, dehydration in a graded series of ethanol to 100% and then critically point dried. The screws were sputter coated with gold and imaged using Zeiss Aurgia FE-SEM, (Carl Zeiss SMT; Thornwood, NY, USA) for qualitative assessment of *S. aureus* colonization of screw retrieved from the revised femurs [44].

## 3. Results

### 3.1. Mechanical Properties of the 3D Printed CaPs

**Figure 2:**
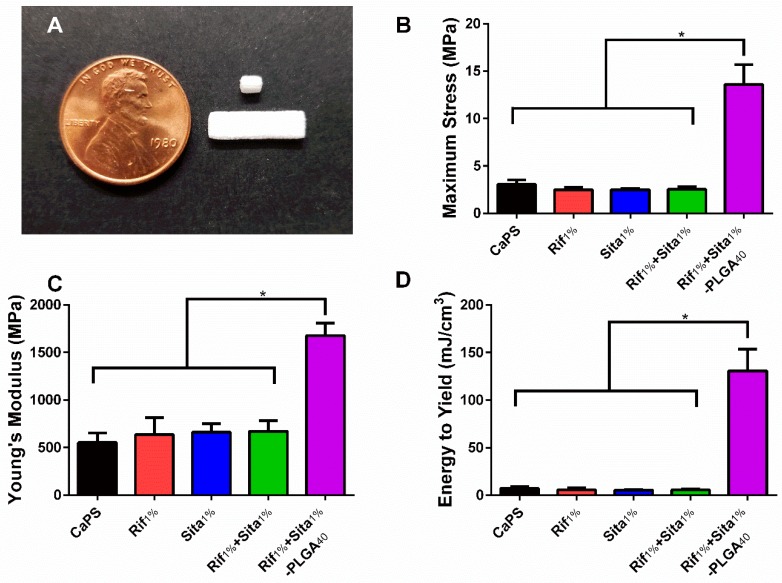
Incorporation of rifampin and sitafloxacin does not affect the material properties of 3D printed calcium phosphate scaffolds (CaPS). (**a**) Ellipsoidal cylinders (1.2 × 2 × 3 mm) were 3D printed by using a modified ZPrinter 450 utilizing a powder bed of 80:20 wt % α-tricalcium phosphate and hydroxyapatitea and a binder solution of 8.75 wt % phosphoric acid supplemented with 0.25 wt % Tween80. Rectangular bars (4 × 1 × 14 mm) were used for flexural testing and cylinders were used for elution studies and implantation into mice. (**b-d**) The addition of rifampin, sitafloxacin, or rifampin and sitafloxacin did not affect the flexural properties (maximum flexural stress, Young’s modulus, and energy to yield) in comparison to CaPS with no additives. However, the addition of two coatings of poly (D,L-lactide-co-glycolide) (PLGA) at 20 wt % and 12.5 wt % for the inner and outer layer, respectively, significantly increased all material properties of maximum flexural stress, Young’s modulus, and energy to yield. CaPS. *N* = 6/group. * denotes *p* < 0.05 determined by Tukey’s post-hoc after ANOVA. Data presented as means ± standard deviations.

Calcium phosphate scaffolds were fabricated using a previously established 3D printing method utilizing a low acidity binder solution [39]. Rifampin, sitafloxacin, and both rifampin and sitafloxacin at 1 wt % were incorporated into the 3D printing process to produce antibiotic containing CaPS (Rif_1%_, Sita_1%_, Rif_1%_ + Sita_1%_). The mechanical properties of the different printed scaffolds were evaluated by 3-point bending. The incorporation of antibiotics into the fabrication process did not affect the maximum flexural stress, Young’s modulus, and energy to yield when compared to CaPS with no incorporated antibiotic (Figure 2B,C). However, biphasic coating of the 3D printed CaPS with an inner layer of 20 wt % PLGA, an outer layer of 12.5 wt % PLGA, and 40 mg/mL of rifampin and sitafloxacin contained in each layer resulted in enhanced biomechanical properties (Rif_1%_ + Sita_1%_-PLGA_40_; Figure 2B,C). Maximum stress and Young’s Modulus were significantly increased 4.4- and 3-fold, respectively, in comparison to CaPS (Figure 2B,C). Additionally, bare scaffolds exhibited brittle fracture resulting in low energy to yield (5.5–7.2 mJ/cm^3^). However, the PLGA coating increased plastic deformation prior to fracture resulting in a significant 17–24-fold increase in energy to yield (Figure 2D). 

### 3.2. Rifampin and Sitafloxacin Release Kinetics from 3D Printed CaPS

**Figure 3:**
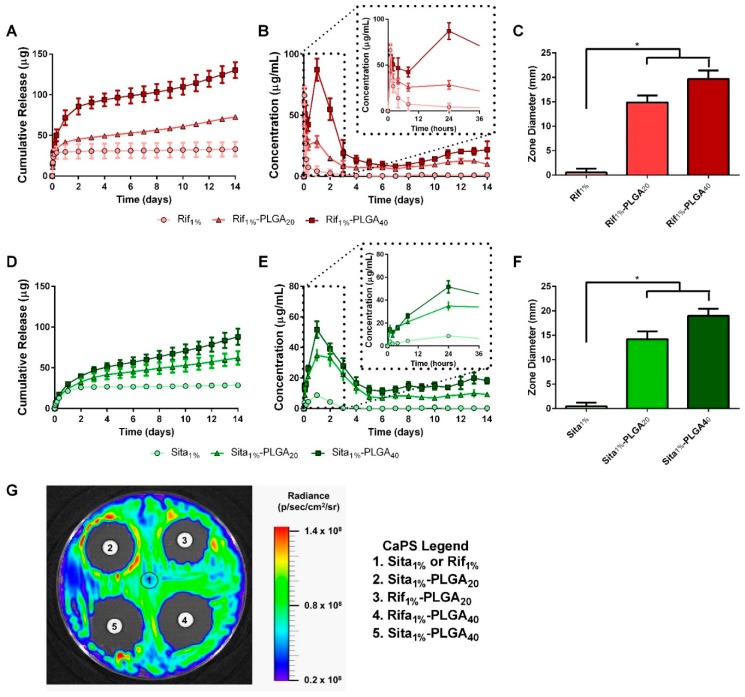
Drug elution kinetics of rifampin- and sitafloxacin-impregnated calcium phosphate scaffolds (CaPS) with PLGA coating. Sitafloxacin and rifampin release profiles were assessed after immersion in PBS for up to 2 weeks (*n* = 5/group). (**a**,**b**,**d**,**e**) Bare scaffolds incorporated with either rifampin (Rif_1%_) or sitafloxacin (Sita_1%_) demonstrated a bolus release within the first 12 h succeeded by no measurable drug concentration by day 3. Coating CaPS with PLGA containing either 20 mg/mL (Sita_1%_-PLGA_20_) or 40 mg/mL (Sita_1%_-PLGA_40_) of drug enabled a biphasic release profile. Initial burst peaking in concentration at 24 h was followed by sustained release exceeding 2 weeks. Drug eluted PBS samples were taken at day 14 to qualitatively and quantitatively measure bioactivity after 2 weeks elution. Eluate was adsorbed overnight by filter paper disks and then placed on a bacterial lawn of Xen36. (**g**) All PLGA coated groups had a distinct zone of inhibition while bare scaffolds had no observed zones. (**c**,**f**) Quantified diameters around the disks significantly increased in PLGA coated groups in comparison to the bare CaPS. *n* = 5/group. * indicates *p* < 0.05 vs. Sita_1%_ or Rif_1%_ by Dunnett’s post-hoc after ANOVA. Data represented as mean ± standard deviation.

The elution of antibiotics from 3D printed CaPS was assessed over two weeks to determine the dose of antibiotics in the 3D printed scaffolds and the effects of biphasic PLGA coating on drug release kinetics. Bare CaPS scaffolds containing either 1 wt % rifampin (Rif_1%_) or 1 wt % sitafloxacin (Sita_1%_) and CaPS with incorporated sitafloxacin or rifampin and additional PLGA coating containing either 20 mg/mL of antibiotic (Rif_1%_-PLGA_20_ or Sita_1%_-PLGA_20_) or 40 mg/mL (Rif_1%_-PLGA_40_ or Sita_1%_-PLGA_40_) were immersed in PBS, and the immersion liquid was changed and sampled at different time points to characterize drug release. Bare scaffolds, Rita_1%_ and Sita_1%_, demonstrated a burst release of >95% of the total cumulative release within the first 12 h (Figure 3B,E). Release from PLGA-coated CaPS with incorporated rifampin or sitafloxacin demonstrated a biphasic dose-dependent release. An initial burst release reaching 70 μg/mL for Rif_1%_-PLGA_40_ and 55 μg/mL for Sita_1%_-PLGA_40_ at 48 h was observed followed by sustained almost zero-order release by the fourth day, which was maintained for two weeks at ~15.0 μg/mL and ~14.4 μg/mL for Rif_1%_-PLGA_40_ and Sita_1%_-PLGA_40_, respectively, which was ~900× minimum inhibitory concentration (MIC) for *S. aureus* (Xen36) [41]. The lower drug dosing in Rif_1%_-PLGA_20_ and Sita_1%_-PLGA_20_ demonstrated a peak concentration that was 33% and 67% of their PLGA_40_ counterparts, respectively, and followed a similar biphasic release profile with sustained release at ~9.1 and ~8.7 μg/mL up to two weeks, which were ~500× MIC for *S. aureus* (Xen36).

The bioactivity of the eluted drugs from the PLGA-coated 3D printed CaPS was also assessed at the end of the two-week study. Filter paper discs were soaked with immersion liquid from day 14 of the study and then placed onto a bioluminescent Xen36 bacterial lawn on TSA. The diameter of the zone of inhibition surrounding the disc was measured (Figure 3G). Both weight concentrations of PLGA-coated CaPS displayed a significant increase in the diameter of their zone of inhibitions in comparison to bare scaffolds (Figure 3C,F). This finding, along with the visible zone of inhibition associated with the PLGA-coated scaffold samples, which was absent for the bare scaffolds, supports the release kinetics data, and further confirms the bioactivity of the drugs eluted from the PLGA-coated scaffolds. Moving forward, the high dose scaffolds (Rif_1%_-PLGA_40_ and Sita_1%_-PLGA_40_) were chosen for subsequent in vivo studies because observed drug release did not reach cytotoxic levels, while providing the highest concentration of sustained drug release [41,45]. Furthermore, scaffolds were also fabricated with both antibiotics, Rif_1%_ + Sita_1%_-PLGA_40_, for in vivo studies to minimize the development of bacterial resistance when delivered as a monotherapy [41,46,47].

### 3.3. In Vivo Efficacy 3D Printed Caps Incorporated with Rifampin and Sitafloxacin 3 Weeks After Post-Revision Surgery

**Figure 4:**
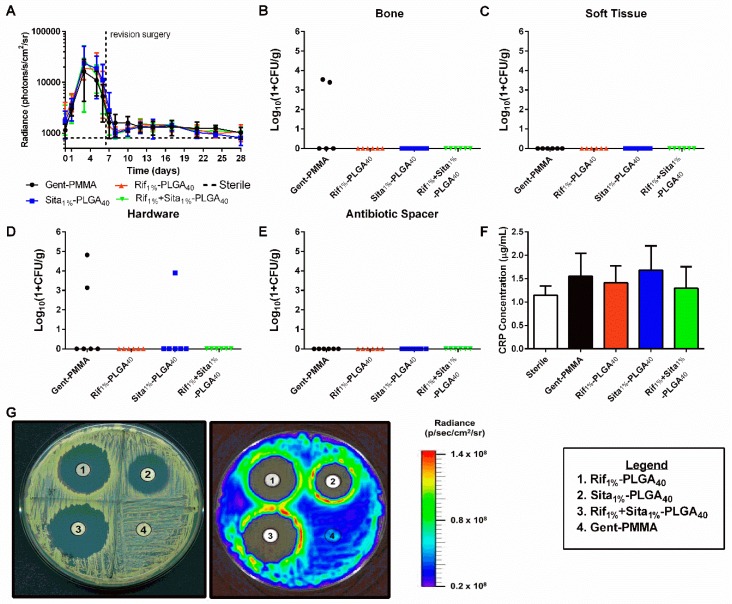
Quantitative assessment of implant-associated osteomyelitis yielded no significant differences between any of the four treatment groups 3 weeks post-revision. (**a**) In vivo longitudinal bioluminescence measurements yielded no differences in bacterial burden between any of the four groups. (**b**–**e**) This is further illustrated by the quantification of bacterial colonization in bone, soft tissue, hardware, and antibiotic spacer 21 days post-revision surgery. 2/5 bone samples for Gent-PMMA were positive for bacterial colonization, 2/6 hardware samples for Gent-PMMA, and 1/6 samples for Sita_1%_-PLGA_40_ were positive for bacterial colonization. All other samples were culture negative. Again, no differences were observed. (**f**) C-reactive protein levels were measured by serum collection. No differences were observed for any group. (**g**) Lastly, CaPS harvested 3 weeks post-revision retained bioactive elution of incorporated antibiotics observed by a zone of inhibition, while Gent-PMMA produced no zone of inhibition. All significance was judged for *p* < 0.05 by 1- and 2-way ANOVA. Bacterial quantification represented as median, all other data was represented as mean ± standard deviation. *N* = 8 samples/group for BLI, *n* = 6 samples/group for CFU, and *n* = 5–8 for CRP quantification.

The in vivo efficacy of antibiotic-laden 3D printed CaPS, Rif_1%_-PLGA_40_, Sita_1%_-PLGA_40_, and Rif_1%_ + Sita_1%_-PLGA_40_ was assessed in a mouse femoral ostectomy model of implant-associated osteomyelitis utilizing a single-stage revision, as previously described [42]. The pathogenic burden was measured longitudinally using BLI. Peak bacterial burden was observed three days post-infection surgery. All groups demonstrated a substantial reduction in BLI after revision surgery and did not increase thereafter. However, no significant differences were observed for any of the four treatment groups. In comparison to the negative control (PMMA cement with no incorporated or systemic antibiotics) of the published study that validated this model, each treatment group in this cohort of mice displayed a significant reduction of BLI 1- and 3-days post-revision as determined by two-way ANOVA with Dunnett’s test (*p* < 0.05) [42]. Quantitative bacterial CFU assays supported the BLI findings indicating no significant differences between any of the groups (Figure 4B,C,E,F). Evidence of persistent infection in bone and bacterial colonization of the implant was only evident in two out of six samples of the Gent-PMMA control (~30% infection persistence rate). Further, the hardware in only one of the Sita_1%_-PLGA_40_ CaPS sample was positive for CFU (Figure 4B,E). The management of infection was further assessed by measuring CRP levels in the serum to assess innate immune response and the onset of inflammation. No significant differences in systemic CRP were observed in any of the three experimental groups and Gent-PMMA in comparison to a sterile control (Figure 4D). 

CaPS and Gent-PMMA harvested three weeks post-revision were sonicated in PBS, and the eluent was used in a Kirby–Bauer disk diffusion assay. All 3D printed CaPS with incorporated antibiotics produced an observable zone of inhibition, which was not observed for the Gent-PMMA spacer (Figure 4E).

### 3.4. In Vivo Efficacy of 3D Printed Caps with Incorporated Rifampin and Sitafloxacin 10 Weeks Post-Revision Surgery

**Figure 5:**
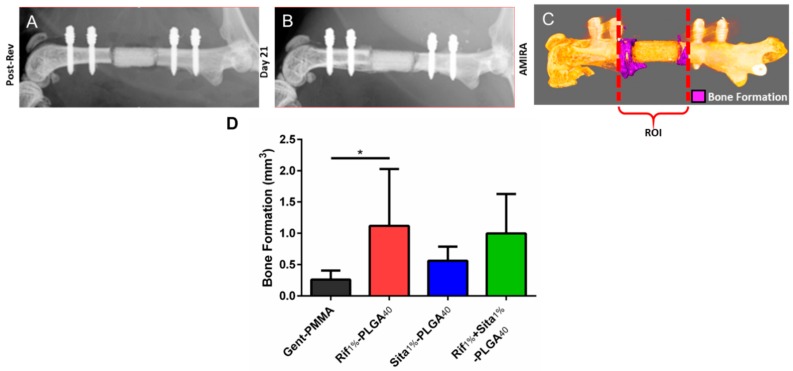
Bone formation following 1-stage revision surgery using gentamicin impregnated PMMA cement spacers (Gent-PMMA) and 3D printed calcium phosphate scaffolds (CaPS) with incorporated rifampin (Rif_1%_-PLGA_40_), sitafloxacin (Sita_1%_-PLGA_40_), and both (Rif_1%_ + Sita_1%_-PLGA_40_). (**a**,**b**) Bone formation was assessed using microcomputed tomography (µ-CT). µ-CT scans were taken post-revision and 21-days posit-revision prior to euthanasia. (**c**) Digital registration was performed using the software AMIRA and an overlay subtraction algorithm was computed to quantify bone formation. (**d**) A significant increase in bone formation was observed for the Rif_1%_-PLGA_40_ in comparison to the clinical control of Gent-PMMA *n* = 4-7/group. * indicates *p* < 0.05 as determined by Dunnett’s post-hoc after ANOVA. Data represented as mean ± standard deviation.

Bone growth within the defect and implanted spacers was assessed through quantitative µ-CT analysis (Figure 5A–C). After three weeks post-revision surgery, a significant increase in bone formation was observed for Rif_1%_-PLGA_40_ in comparison to the clinical control Gent-PMMA (Figure 5D). 

**Figure 6:**
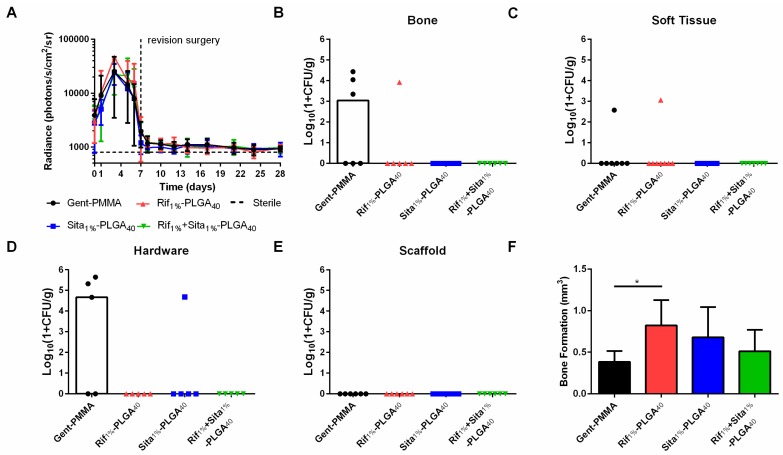
Quantitative assessment of infection management and bone regeneration 10 weeks post-revision surgery for 3D-printed rifampin- and sitafloxacin-laden-CaPS in a mouse model of implant-associated osteomyelitis. Implant-associated osteomyelitis yielded no significant differences between any of the four treatment groups regarding infection management; however, a significant increase in bone formation was observed after 10 weeks of healing. (**a**) In vivo longitudinal bioluminescence measurements yielded no differences in bacterial burden between any of the four groups up to 3 weeks post-revision surgery. (**b**–**e**) This is further illustrated by the quantification of bacterial colonization in bone, soft tissue, hardware, and antibiotic spacer 10 weeks post-revision surgery. Again, no differences were observed, however, as 3/6 and 1/6 bone samples were culture positive for the clinical control and Rif_1%_-PLGA_40_, respectively. 3/5 and 1/5 hardware samples were culture positive for Gent-PMMA and Sita_1%_-PLGA_40_ respectively. 1/7 soft tissue samples were culture positive for both Gent-PMMA and Rif_1%_-PLGA_40_. (f) Lastly, significant bone healing was observed in Rif_1%_-PLGA_40_ in comparison to the clinical control. All significance was judged for *p* < 0.05 by 1- and 2-way ANOVA. Bacterial quantification represented as median, all other data was represented as mean ± standard deviation. *N* = 8 samples/group for BLI, *n* = 5–8 samples/group for CFU and µCT-quantification.

This observation motivated an extended study in which additional cohorts of mice were assessed at 10 weeks post-revision to assess long-term bone healing. Here again, no differences were observed for longitudinal BLI monitored up to three weeks post-revision surgery (Figure 6A). At 10 weeks post-revision, CFU quantification reflected similar outcomes to what was observed at 3 weeks post-revision. Half of the Gent-PMMA control bone samples were culture positive for Xen36 (Figure 6B) and only one of the Rif_1%_-PLGA_40_ bone samples were positive (Figure 6B). Similarly, three hardware samples in the Gent-PMMA control group were colonized by Xen36. In contrast, Sita_1%_-PLGA_40_ was the only other group that had a sample in which the hardware was CFU positive (Figure 6D). Lastly, a significant increase in bone formation was observed for Rif_1%_-PLGA_40_ in comparison to Gent-PMMA, 10 weeks post-revision surgery (Figure 6F), which did not appear to be significantly increased compared to the bone formation measured at 3 weeks. 

**Figure 7:**
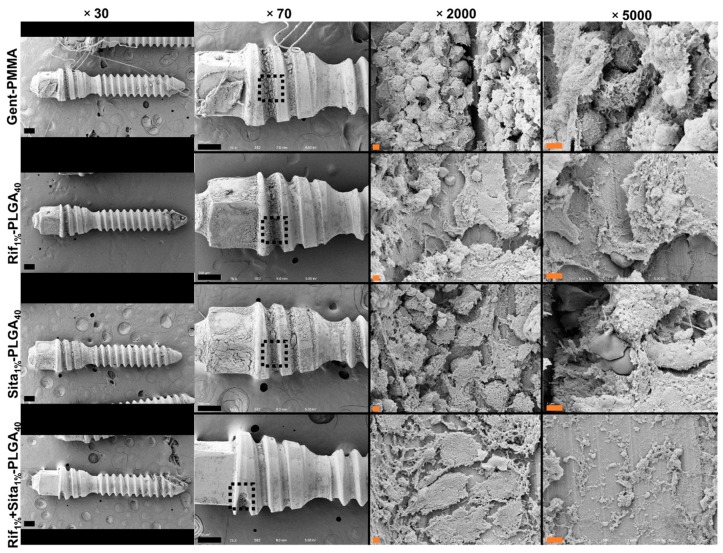
Representative scanning electron microscopy (SEM) of harvested hardware after 10 weeks post-revision surgery. Titanium screws were harvested 10 weeks post-revision surgery to qualitatively investigate biofilm formation and the presence of *Staphylococcus aureus* colonization between groups. Representative images are shown at magnifications of 30×, 70×, 2000× and 5000×. No structures consistent with 1 µm diameter of *S. aureus* cocci were observed in any of the four groups. Black scale bars indicate 200 µm and orange scale bars indicate 2 µm.

SEM was utilized to thoroughly examine the topography of harvested titanium screws at 10 weeks post-revision for the presence of biofilm or bacterial colonization. Qualitatively assessing the screws revealed no evidence of either biofilm presence or planktonic *S. aureus* cells (Figure 7). 

Lastly, at 10 weeks post-revision, 3D printed Rif_1%_-PLGA_40_, Sita_1%_-PLGA_40_, Rif_1%_ + Sita_1%_-PLGA_40_, and Gent-PMMA were harvested, placed into PBS, sonicated, and the eluent used for a Kirby–Bauer disk diffusion assay. However, no zone of inhibitions were observed (data not shown).

## 4. Discussion

The recalcitrant and recurrent nature of *S. aureus*-related implant-associated osteomyelitis continues to be an onerous burden for orthopedic surgery. The current clinical standard of treatment relies on antibiotic-laden PMMA bone cement that enables high concentrations of antibiotic to be delivered to the infection site. Currently only five bone cement products are Food and Drug Administration (FDA)-approved for antibiotic delivery; Stryker’s Simplex P which contains tobramycin; Zimmer’s Palacos G, which contains gentamicin; Depuy Orthopaedic’s high and low viscosity SmartSet, which contain gentamicin; and DePuy’s Postalac prosthesis containing tobramycin and vancomycin [48,49]. However, due to the limited choice of antibiotics as well as the increased commercial costs, clinicians often manually mix antibiotics with PMMA powder at varying ratios [50]. The various antibiotics incorporated into PMMA bone cement powder as well as the different mixing procedures and additives lead to vastly variable and insufficient drug release kinetics and clinical outcomes [31,51,52,53]. Furthermore, PMMA is non-biodegradable, requires a removal surgery, and can also be a nidus for bacterial colonization, despite thorough surgical debridement [54,55]. These various shortcomings, as well as the lack of a universally-accepted intraoperative procedure for using antibiotic-laden PMMA bone cement motivate the search for alternative therapeutic solutions, such as the use of biodegradable and osteoconductive spacers to serve the dual purpose of local antibiotic delivery and subsequent stimulation of bone regeneration. 

Biocompatible and osteoconductive CaPS were 3D printed with either rifampin, sitafloxacin, or in combination using a previously established method [36]. Rifampin is a rifamycin-class antibiotic, which inhibits bacterial DNA transcription [56]. Because this action is independent of cellular division, rifampin has proven potent activity against established *S. aureus* biofilm [40]. The use of rifampin is popular in biofilm-associated infections, however its local delivery in orthopedic applications is hindered by its inability to be incorporated into PMMA bone cement, since it acts as a free radical scavenger and interferes with PMMA polymerization [35,40,57]. Thus, the use of 3D printed CaPS as an alternative spacer biomaterial overcomes this limitation of PMMA and allows the incorporation of rifampin for local delivery. In addition, to rifampin, we recently showed that sitafloxacin has the potential for local management of orthopedic infections due to its potent activity against biofilm and small colony variants [41]. Sitafloxacin is a fluoroquinolone antibiotic that inhibits bacterial type II DNA topoisomerase [58]. Despite the proven and promising applications of rifampin and sitafloxacin, this study is the first to examine the individual and concomitant local delivery of these two antibiotics from 3D printed CaPS for the treatment of orthopedic implant-associated osteomyelitis in a mouse model.

Characterization of the mechanical properties of CaPS with and without incorporated antibiotics yielded no changes in mechanical strength; however a caveat to using CaP as a scaffold for large bone defects is its relatively low mechanical strength and brittle nature (Figure 2; 3 MPa max stress, 7 mJ/cm3 energy to yield). To compensate for this, the addition of a biphasic PLGA coating both enhanced mechanical strength and provided ductility to the material properties. PLGA has widely been used in different applications for drug delivery due to its biocompatibility and degradability into glycolic and lactic acid in the body and is approved by the FDA for clinical application [59,60]. Furthermore, it has been implemented in numerous systems for successful bone regeneration [61,62,63,64]. Yet, with the added PLGA, flexural strength is still not within the range of trabecular bone (20–45 MPa). However, when compared to other 3D printed CaP composite scaffolds, these scaffolds fall within the range of published flexural strength (e.g., ~70 kPa flexural strength for CaP-collagen (1.5 wt %) scaffold [39], ~10 MPa flexural strength for CaP-alginate (2.5 wt %) scaffold [65], 1.27 MPa flexural strength for HA/apatite-wollastonite glass composite [66], 5.2 MPa flexural strength for brushite scaffold [67], 3.5 MPa flexural strength for CaP-polycaprolactone (1 wt %) composite scaffold [68]). Other 3D printed CaP scaffolds have achieved mechanical strength comparable to both cortical and trabecular bone (30 MPa flexural strength and 110 MPa compressive strength for Sr-HT (Sr doped Ca2ZnSi2O7(HT))-Granite scaffolds [69], 50 MPa flexural strength for HA/bis-GMA scaffolds [70]). However, these scaffolds require prolonged high-temperature sintering, which is not compatible with incorporation of bioactive drugs such as antibiotics. Therefore, a PLGA coating that maintains the biocompatible and osteoconductive nature of these scaffolds [63,64], enhances mechanical strength, and enables prolonged and sustained drug release was used. 

Characterization of the drug release kinetics of antibiotic-impregnated PMMA bone cement spacers demonstrate major discrepancies from commercially available antimicrobial PMMA products and also hand-mixed antibiotic cements. In vitro studies generally show that less than 10% of the total incorporated antibiotic drug is released, occurring in a burst mechanism, typically within the first 3–7 days [31,51,52]. Herein, we demonstrated that the drug release from our 3D printed CaPS is biphasic in nature, first demonstrating an initial burst release, followed by a prolonged and sustained release up to two weeks in vitro. This is beneficial because sustaining local drug concentrations above MIC values is important for the preemption of the emergence of resistant colonies [71]. 

To test the efficacy of rifampin- and sitafloxacin-laden CaPS, an appropriate mouse model was carefully considered to best represent clinical scenarios and enable clinically translatable results. Previously, our group has developed and implemented a mouse model of implant-associated osteomyelitis that produces hallmark features such as biofilm formation and osteolysis, which required debridement for spacer implantation and assessment [72]. In this original model, the fixation plate and screws used to stabilize the plate were retained. In a study investigating the performance of both systemic antibiotics and locally delivered vancomycin and rifampin via 3D printed CaPS using this model, quantitative outcome measures such as longitudinal BLI and quantitative assessment of end point CFUs were reduced, however bacterial colonization persisted within the bone and on fixation hardware [36]. This study exemplifies the caution surrounding hardware retention in clinical scenarios, where relapses in infection have been reported to be as high as 54% due to biofilm on implant surfaces [13]. Thus, surgical revisions, where the hardware is completely exchanged, remain the gold standard. To mimic this practiced surgical method, we chose to modify the mouse model to enable a revision surgery with complete hardware removal and exchange after establishment of infection. 

In this model, systemic vancomycin treatment and local delivery of antibiotics via PMMA bone cement or 3D printed CaPS nearly eradicated the bacterial bioburden. Regardless of the incorporated drug, 3D printed CaPS exhibited efficient management of implant-associated osteomyelitis, equivalent to the clinical control (Gent-PMMA) as demonstrated by longitudinal BLI, immune response as measured by CRP, and qualitative absence of biofilm measured at both 3- and 10-weeks post-revision. However, reduced bacterial colonization rates, as measured by quantified CFUs, were observed 3- and 10-weeks post-revision. The results demonstrated here display superior outcome measures in comparison to similar small animal models that retain orthopedic hardware [36,73]. These results validate the logic that hardware exchange is necessary to prevent possible relapse in infection; however orthopedic surgeons debate whether a 1- or 2-stage revision is necessary. Meta-analyses comparing the clinical failure rates of 1- and 2-stage revisions have determined similar reinfection rates, indicating rates of 7.6% and 8.8% respectively [74]. However, 2-stage revisions require an extra surgery for bone cement removal resulting in higher likelihood for damaging surrounding bone soft and soft tissue as well as requiring more time and costs, yet 2-stage revisions still remain the most commonly performed [14,15,75,76]. One practical alternative would be to replace the PMMA cement used in 2-stage revisions with a biocompatible and osteoconductive scaffold, which could be retained to eliminate the need for a second surgery. In this study, the critical finding was that CaPS incorporated with antibiotics not only improved the management of infection, but rifampin laden CaPS also significantly enhanced new bone formation in comparison to PMMA spacers. Interestingly, this is consistent with previous reports. Shiels et al. [77] demonstrated that the direct addition of rifampin powder to a contaminated wound led to greater callus formation when compared to untreated wounds, while vancomycin powder elicited no effect. Insignificant bone formation of CaPS containing sitafloxacin also supports studies reporting negative effects of fluoroquinolones on fracture healing [78,79]. Although significant bone formation was observed in this study, no additional bone growth between 3 and 10 weeks occurred suggesting that these 3D printed CaPS with incorporated antibiotics, while osteoconductive, are not osteoinductive. The addition of osteoinductive elements are needed to further enhance bone healing with these scaffolds in order to attain the objective of osseous bone bridging of the segmental defect. Hence, a limitation to both CaPS and PLGA is that, although osteoconductive, these materials lack osteoinductivity. However, these materials have been shown to be compatible with a range of osteoinductive components such as bone morphogenetic protein (BMP)-2, vascular endothelial growth factor (VEGF), transforming growth factor (TGF)-β, fibroblast growth factor-2, and mesenchymal stem cells (MSC) enabling significant bone defect repair [80,81,82,83,84,85,86,87,88]. Addition of such osteoinductive elements is needed to further enhance the bone healing potential of these scaffolds in order to attain the objective of osseous bone bridging of the septic segmental defect. To date, no study has demonstrated the healing of a septic critical-size defect; however, we believe this study serves as the foundation for the addition of osteoinductive elements to our antibiotic-laden CaPS that will enable the accomplishment of this objective.

One limitation to this study is the usage of a MSSA strain of bacteria over a MRSA strain. Orthopedic infections caused by MRSA are more difficult to treat and virulent, which leads to longer hospital stays, increased number of staged surgeries, and higher treatment failure and mortality rates than those caused by MSSA [89,90,91]. This in part may explain the excellent infection management demonstrated by the 3D printed CaPS with incorporated rifampin and sitafloxacin. Future studies will determine the efficacy of using a 1-stage revision with 3D printed CaPS with rifampin and sitafloxacin to treat an implant-associated MRSA infection, using both laboratory strains and clinical isolates. Although no significant bone formation was observed for 3D printed CaPS with sitafloxacin, its advantage is its potency against persister cells such as SCV. Future work will incorporate stable strains of SCV into this model to elucidate this effect. Forthcoming studies will also incorporate proven osteoinductive elements, such as mesenchymal stem cells, bone morphogenetic protein-2, or demineralized bone matrix, into the 3D printing process to further enhance bone healing potential [92,93,94].

## 5. Conclusions

This study showcases the therapeutic efficacy of performing a 1-stage revision with complete hardware exchange in a preclinical mouse model of *S. aureus* osteomyelitis treated with 3D printed CaPS incorporated with rifampin and sitafloxacin in comparison to a 2-stage revision first treated with gentamicin-laden PMMA and then CaPS. Effective infection management and reduced bacterial colonization rates of the 3D printed scaffolds were attributed to the biphasic local delivery of antibiotics achieved by PLGA coatings. Furthermore, the biocompatibility of the 3D printed CaPS, enabled significant bone formation. However, no additional growth between 3- and 10-weeks post-revision indicate the need for additional bioactive factors to increase bone healing. Future studies will incorporate osteoinductive elements to further enhance bone healing and possible full bone regeneration.
